# 影像组学在肺癌检查点抑制剂相关肺炎诊疗中的研究进展

**DOI:** 10.3779/j.issn.1009-3419.2026.101.17

**Published:** 2026-07-20

**Authors:** Dong ZHANG, Li PENG, Tianming ZHANG, Fanqi WU, Hong WANG

**Affiliations:** 730000 兰州，兰州大学第二医院呼吸与危重医学科; Department of Pulmonary and Critical Care Medicine, Lanzhou University Second Hospital, Lanzhou 730000, China

**Keywords:** 肺肿瘤, 影像组学, 检查点抑制剂相关肺炎, Lung neoplasm, Radiomics, Checkpoint inhibitor-related pneumonitis

## Abstract

免疫检查点抑制剂（immune-checkpoint inhibitors, ICIs）可显著改善肺癌患者的预后，但检查点抑制剂相关肺炎（checkpoint inhibitor-related pneumonitis, CIP）作为最严重的免疫相关不良事件之一，仍缺乏明确的诊断标准和可靠的风险分层工具。影像组学能够从计算机断层扫描图像中提取高通量特征，为CIP的早期识别和风险分层提供了一种无创诊断技术手段。本文综述了影像组学在肺癌免疫治疗所致CIP风险预测、诊断与鉴别诊断、预后评估、多组学发病机制及可解释性人工智能等方面研究的最新进展，以期为其临床应用提供依据。

## Abstract (en)

Immune checkpoint inhibitors (ICIs) have significantly improved the prognosis of patients with lung cancer. However, checkpoint inhibitor-related pneumonitis (CIP), as one of the most severe immune-related adverse events, lacks well-defined diagnostic criteria and reliable risk stratification tools. Radiomics enables high-throughput feature extraction from computed tomography images and provides a non-invasive technical approach for the early identification and risk stratification of CIP. This article systematically reviews the recent advances in the application of radiomics to risk prediction, diagnosis and differential diagnosis, and prognostic evaluation of CIP in lung cancer immunotherapy. Furthermore, it explores the value of integrating radiomics with multi-omics data in elucidating the pathogenesis of CIP, as well as the role of explainable artificial intelligence (XAI) in enhancing the clinical trustworthiness of models.

肺癌是全球发病率最高的恶性肿瘤，约占所有癌症的12.4%^[1]^。免疫检查点抑制剂（immune-checkpoint inhibitors, ICIs）改变了肺癌的治疗格局，但其广泛应用伴随着免疫相关不良事件发生率的增加，其中检查点抑制剂相关肺炎（checkpoint inhibitor-related pneumonitis, CIP）是最严重的事件之一^[2]^。CIP患者常表现为咳嗽、呼吸困难和低氧血症等临床症状，重症患者可迅速进展为呼吸衰竭甚至死亡。虽然CIP的总体发生率低^[3]^，但一旦发生，需要根据严重程度暂停或终止免疫治疗。同时，CIP的排除性诊断常导致识别与干预延迟，严重影响患者预后。

影像组学从计算机断层扫描（computed tomography, CT）图像中提取高通量特征，将细微的影像学信息转化为可量化的数据，其基本流程包括图像获取与预处理、感兴趣区域（region of interest, ROI）分割、特征提取、特征筛选与模型构建。它在肺炎病因鉴别、肺癌早期诊断和治疗反应预测方面取得了显著进展，为CIP后续研究提供依据^[4]^。此外，可解释性人工智能（explainable artificial intelligence, XAI）技术能可视化深度学习影像组学模型的决策依据，有效解决“黑箱”问题，提高临床可信度和转化潜力。本文总结影像组学在CIP风险预测、鉴别诊断与预后评估中的最新应用，指出影像组学在激素耐药性CIP中应用的可行性，同时探讨多组学整合与XAI技术在机制阐明和模型可信度提升方面的潜力，并分析当前研究的共性缺陷与临床转化障碍，以期为CIP的精准诊疗提供参考。

## 1 CIP的影像学特征与分类

CIP的影像学表现包括磨玻璃影、实变、网状影和小叶间隔增厚，以磨玻璃影最为多见^[5]^。基于上述影像学特征，CIP通常分为5种类型：机化性肺炎、过敏性肺炎、非特异性间质性肺炎、急性间质性肺炎/弥漫性肺泡损伤和普通型间质性肺炎样类型^[6]^。其中，机化性肺炎最常见，而弥漫性肺泡损伤预后最差^[7]^。

除基于影像学分型外，CIP还可从发病时间和治疗反应两个维度进一步分层。在发病时间方面，Huang等^[8]^将CIP分为早发型和晚发型，发现早发型以机化性肺炎居多，晚发型则常表现为非特异性间质性肺炎，且早发型患者严重程度更高、总生存期更差（P<0.05）。上述结果提示，发病时间亚型间存在细微影像学差异，影像组学有望量化这些差异。在治疗反应方面，CIP确诊后，2级及以上患者需立即停用ICIs并启动糖皮质激素治疗^[9]^。根据对激素治疗的反应，CIP可分为激素无反应性或激素依赖性亚型。激素无反应性包含激素难治性和激素抵抗性两类亚型，前者指对糖皮质激素治疗无反应；后者指对激素有部分反应，但未完全缓解。激素依赖性CIP是指对初始激素治疗有反应但在激素减量期间复发，激素无法减停^[10,11]^。研究^[12]^显示，激素难治性CIP以弥漫性肺泡损伤和机化性肺炎多见，患者合并间质性肺病比例更高，且更易发展为3-4级CIP^[13]^。另有研究^[14]^表明，激素难治性CIP会降低肺癌患者的1年生存率，提示预后不良，且免疫调节剂对该类患者仅部分有效^[15,16]^。后续研究可利用影像组学早期识别激素抵抗性CIP高风险患者，以便加强监测和抢先干预。表1总结了CIP分型影像学、临床特征及预后治疗对比。

**表1:** CIP分型影像学、临床特征及预后治疗对比

Classification	Subtype	CT manifestations	Clinical characteristics	Corticosteroid response	Prognosis
Image	OP	GGO, consolidation with air bronchogram	Most common	Sensitive	Inferior
	HP	Diffuse centrilobular ground-glass nodules	Favorable prognosis	Sensitive	Superior
	NSIP	Basilar reticulation, interlobular septal thickening	Chronic fibrosis	Dependent	Superior
	AIP/DAD	Bilateral diffuse consolidation, extensive GGO	Rapid deterioration	Refractory	Worst
Onset time	Early-onset	OP pattern	Rapid progression	NR	Inferior
	Late-onset	NSIP pattern	Slow	NR	Superior
Steroid-response	Steroid- unresponsive	DAD pattern	Additional immunosuppression	No/partial improvement	Inferior
	Steroid-dependent	NSIP pattern	Relapse	Impossible to stop	Inferior

CIP: checkpoint inhibitor-related pneumonitis; CT: computed tomography; AIP/DAD: acute interstitial pneumonia/diffuse alveolar damage; GGO: ground-glass opacities; HP: hypersensitivity pneumonitis; NR: not reported; NSIP: non-specific interstitial pneumonia; OP: organizing pneumonia.

综上所述，CIP的影像学与临床分型体系日趋细化，影像组学作为一种高通量的图像定量分析技术，有望将上述分型标准中隐含的细微影像差异转化为可量化的生物标志物，为CIP的风险预测与个体化治疗决策提供客观依据。

## 2 影像组学在CIP管理中的应用

### 2.1 预测CIP发生

确诊CIP是一个排除性诊断过程，从症状出现到启动皮质类固醇治疗需要1-9天^[17]^。在此期间，CIP可能迅速进展为呼吸衰竭甚至死亡，严重影响患者预后^[18]^。因此，预测CIP风险、对高危个体进行密切随访具有重要意义。血清生物标志物如白细胞介素（interleukin, IL）-6、IL-10和中性粒细胞与淋巴细胞比率与CIP风险相关^[19]^，但特异性有限。因此，利用影像组学预测CIP发生并关注高风险个体已成为一个主要研究焦点。

基于ROI的划分方式，现有预测研究可分为3类：全肺、肺实质（剔除肿瘤及血管组织）、肺癌病灶的特征提取研究。Tan等^[20]^纳入24例CIP患者与24例匹配对照者，融合两阶段迁移学习与对比学习方法构建深度学习影像组学预测模型，受试者工作特征曲线下面积（area under the curve, AUC）达0.918。该研究采用五折交叉验证弥补样本量偏小的缺陷，但外部泛化能力仍需大样本独立队列验证。相较于单一影像特征提取方式，多特征融合提取策略可有效提升模型预测性能：一研究融合影像组学特征与深度学习特征构建多模态模型，其AUC达0.829^[21]^。此外，多项研究^[22-24]^均证实了上述结论，如Lyu等^[23]^融合深度学习特征、影像组学特征及临床特征，采用半自动ROI标注，AUC达0.895；Wang等^[24]^则开发全自动深度学习影像分析模型，AUC为0.80。两项研究ROI勾画方式各有优势，半自动联合人工修正的ROI勾画方式可取得较高的预测精度，而全自动勾画因其简便性及可重复性在临床推广中更具优势。尽管上述研究证明联合模型性能更优，但多特征融合需警惕过拟合风险。鉴于CIP发生于非肿瘤区域肺组织，Cousin等^[25]^分别从肺叶、全肺两个解剖层面提取影像特征构建预测模型，发现肺叶层面模型的预测性能略优，但差异无统计学意义，这可能与研究样本量不足有关，仍需大规模队列研究加以验证。为精准预测非小细胞肺癌（non-small cell lung cancer, NSCLC）患者免疫疗效及相关不良反应，Yadav等^[26]^以肿瘤原发病灶及病灶周围1 cm范围内的肺实质作为ROI，探究肿瘤本体及肿瘤微环境的影像学细微改变对CIP发生的预测价值，但AUC仅为0.6，提示单纯依赖肿瘤病灶的影像组学特征可能不足以有效预测CIP。综合上述研究，全肺/肺实质ROI勾画策略总体优于肿瘤ROI，可能与CIP的病理本质有关——CIP是免疫细胞攻击正常肺组织引发的弥漫性炎症损伤，而非局限于肿瘤局部，因此全肺ROI能更全面地捕捉炎症相关的影像改变。表2^[20-26,42,43]^总结了利用影像组学技术进行CIP预测研究的主要特征。

**表2:** CIP预测的影像组学模型总结

Study	Sample	ROI	Model	Visualization	AUC (95%CI)	Limitation
Tan et al ^[20]^	24/48	Whole lung	CNN	CAM	0.92 (NR)	Small sample
Smith et al ^[21]^	162/671	Whole lung	RF	None	0.83 (0.78-0.88)	High false-positive rate
Cheng et al ^[22]^	40/141	Whole lung	LR	None	0.90 (0.72-1.00)	Low PPV
Lyu et al ^[23]^	51/92	Whole lung	LR	None	0.89 (NR)	Retrospective
Wang et al ^[24]^	77/157	Whole lung	SVM	Grad-CAM	0.80 (0.68-0.91)	No external validation
Cousin et al ^[25]^	35/116	parenchyma	RF	None	0.75 (0.64-0.86)	Heterogeneous patients
Yadav et al ^[26]^	31/159	Tumor	RF	None	0.60 (0.55-0.66)	Poor performance
Chen et al ^[42]^	3/85	Tumor	LCI-RPV	None	0.74 (0.64-0.84)	Small sample
Delasos et al ^[43]^	56/293	Whole lung	LR	None	0.89 (0.83-0.95)	Limited generalization

ROI: region of interest; Grad-CAM: gradient-weighted class activation mapping; PPV: positive predictive value; RF: random forest; SVM: support vector machine; AUC: area under the curve.

总体而言，整合影像组学、深度学习和临床特征的多模态模型预测效能普遍优于单一模态模型；深度学习提取的特征可有效补充传统影像组学特征信息，提升模型预测效能；ROI勾画的最佳策略尚未统一，且无三者对比研究，未来需进一步探索，明确不同ROI策略的优劣及适用场景。

### 2.2 辅助CIP诊断与鉴别诊断

风险预测模型筛选高危人群可指导后续随访，患者出现疑似CIP的临床症状，首要问题便是与肺部感染、放射性肺炎等疾病相鉴别。这一临床过程仍具有挑战性：CIP与肺部感染、放射性肺炎（radiation pneumonitis, RP）和其他治疗相关性肺炎（other treatment-related pneumonitis, OTP）重叠的临床表现；疾病间高度相似的影像特征；免疫联合放疗增加诊断复杂性。应用影像组学有助于解决上述临床困境，实现早诊断、早治疗。

Tohidinezhad等^[27]^开发影像组学模型来区分CIP和OTP（CIP 31例，OTP 41例），从75 mm球形ROI半自动提取特征，经LASSO-LR筛选3个特征构建模型，AUC达0.83，优于临床模型（AUC=0.66）。但研究未纳入III期患者且样本量受限导致模型外推性不足。有证据^[28]^表明III期患者的CIP发生率更高，此类患者接受放化疗联合免疫治疗，放射诱导的肺损伤会产生更复杂的影像模式。因此，鉴别CIP与放射性肺炎具有更重要临床意义，Hindocha等^[29]^在多中心队列（CIP 103例，RP 111例）中提取4个影像组学特征构建鉴别分类器，训练集和验证集中的AUC分别为0.77、0.8。然而，模型性能在放免联合亚组中显著下降（AUC=0.58），提示该亚组存在高度相似的影像特征或特殊混合表型。Wang等^[30]^针对上述空白，在30例联合治疗患者中筛选4个关键鉴别特征：小波LLL-灰度共生矩阵_联合均值的存在提示RP，而其余3个特征（原始影像-灰度游程矩阵-长行程低灰度级侧重；小波HLL-一阶统计量-中位数；小波LLL-邻域灰度差矩阵-活跃度）则倾向于CIP。这说明小波变换特征可捕捉多尺度纹理信息，或提升模型在联合治疗亚组中的稳健性；尽管未探讨这些特征的生物学相关性，但分类器在区分CIP和RP方面表现出了良好的诊断性能。表3^[27,29,30]^总结了利用影像组学进行CIP诊断的研究的核心特征。

**表3:** CIP诊断的影像组学模型总结

Study	Sample (CIP/Control)	Feature	Model	Characterization	AUC (95%CI)
Tohidinezhad et al ^[27]^	31/41	RAD+CL	LR	75-mm spheroidal ROI	0.83 (0.77-0.95)
Hindocha et al ^[29]^	103/111	RAD	LR	Four category discrimination	0.80 (0.64-0.92)
Wang et al ^[30]^	50/50	RAD+CL	LDA, RF	Clustering analysis	0.85 (0.70-1.00)

CL: clinical; LDA: linear discriminant analysis; RAD: radiomics.

当前鉴别诊断的核心挑战在于CIP与RP的影像重叠，特别在联合治疗人群中。现有研究在单纯CIP与RP鉴别中表现良好，在联合治疗人群中性能显著下降，说明传统影像组学模型不足以区分混合影像模式，未来研究可引入生境分析、深度学习等方法，以提升模型在该复杂场景中的鉴别能力。

### 2.3 评估CIP预后

CIP特别是重症CIP，严重影响患者预后，亟需可靠的预后生物标志物来指导风险分层和个体化管理。既往研究^[31]^已使用血清标志物和临床特征进行预后评估。例如，Wang等^[32]^发现CIP发病时间、病灶与肿瘤的位置关系、网状影的存在及球蛋白水平均为独立预后因素；病灶远离原发肿瘤（P=0.023）和无网状改变（P=0.013）的CIP患者生存期更长。随着影像组学技术的成熟，Balbi等^[33]^利用CT影像组学特征的纵向变化（Δ-radiomics）联合全身炎症指标，构建晚期NSCLC患者生存期预测模型，一致性指数达0.75。传统的单时点影像组学仅能反映某一时刻的影像特征，无法捕捉CIP发生发展过程中的动态变化；Δ-radiomics通过比较不同时间点影像组学特征的变化量，可反映疾病演进过程中的影像演变轨迹，同时可识别炎症负荷的动态变化趋势，预判重症化风险。针对CIP预后评估的影像组学研究较少，未来的研究应整合动态影像组学特征与相关的临床和血液学指标，构建稳健的CIP预后模型，以便更好地进行早期管理和预后评估。

除CT提供的结构信息外，正电子发射计算机断层显像/CT（positron emission tomography/CT, PET/CT）所反映的代谢活性为CIP预后评估提供了重要的功能维度补充。PET/CT成像原理在于肿瘤细胞和代谢活跃的炎症细胞对^18^F-氟代脱氧葡萄糖（^18^F-fluoro-2-deoxy-D-glucose, ^18^F-FDG）的高摄取，探测器捕获正电子衰变产生的湮没光子后生成功能图像，再与CT解剖图像融合，从而呈现病变的代谢信息和精确位置^[34]^。CIP的发生与肺部可能已存在的慢性炎症或免疫激活状态有关，PET/CT能将这种“预存炎症”可视化并量化。有研究^[35]^发现，外周肺^18^F-FDG标准摄取值最大值（standardized uptake value maximum, SUVmax）>1.1可有效预测CIP的发生（AUC=0.72, P<0.01），且高外周SUVmax是重度肺炎和总体免疫相关不良事件（immune-related adverse events, irAEs）的独立预测因子。然而，一项纳入239例肺癌患者的研究^[36]^得出了矛盾的结论：非肿瘤肺区域的高FDG摄取不能预测CIP的发生。该研究仅分析了明确界定的非肿瘤区域，排除了肺转移和炎症区域等混杂因素，并计算了4个球形区域的平均SUV，侧重于全肺的整体代谢水平，可能稀释局部高代谢信号；而Kim等^[35]^则关注对侧外周非肿瘤肺组织的SUVmax，并且纳入了更高比例的慢性肺部疾病病史患者，更易捕捉炎症信号，这可能解释了两者结论的差异。鉴于单一SUV指标预测结果的不一致，后续研究可以结合临床、血清学因素和影像组学高阶纹理特征来构建多模态模型以提高预测性能，同时开展多中心、大样本研究以明确PET/CT在CIP预后评估中的确切价值。

## 3 多组学用于CIP管理

CIP的发病机制尚未完全阐明，主要的假说涉及T细胞活性增强和炎症细胞因子信号通路失调^[37]^。活化的T细胞可能因交叉抗原攻击正常肺组织^[37,38]^，促炎细胞因子，如肿瘤坏死因子（tumor necrosis factor, TNF）-α和IL-6，引发细胞因子风暴进一步加重组织损伤^[19,39]^。除T细胞及相关细胞因子参与CIP的发病^[37,40]^，CIP患者B细胞和巨噬细胞的表达也显著增加，这提示新治疗靶点可能，从而进一步阐明CIP的启动机制^[41]^。此外，基因组学和转录组学通过基因集富集分析（gene set enrichment analysis, GSEA）帮助识别与CIP相关的信号通路。

影像组学特征与上述生物学过程之间的关联，可通过基因组学与转录组学分析初步阐明。Chen等^[42]^整合了基因组学和影像组学，基于NSCLC患者的增强CT图像和CD274计数[程序性死亡配体1（programmed death ligand-1, PD-L1）蛋白编码基因RNA表达水平]，构建了肺癌免疫治疗影像组学预测向量（lung cancer immunotherapy-radiomics prediction vector, LCI-RPV），在PD-L1抑制剂亚组中预测CIP的AUC达0.74；但该研究仅纳入3例CIP患者，存在过拟合风险。此外，该研究的主要终点是预测PD-L1抑制剂治疗反应而非CIP，结论需谨慎解读。Delasos等^[43]^开发了一个临床病理-影像组学列线图用于预测CIP（AUC=0.89），确定了Law纹理特征与CIP发展有关后，通过GSEA分析发现高CIP风险与细胞外基质-受体相互作用、T细胞受体信号传导、细胞因子-细胞因子受体相互作用等促炎及免疫激活通路相关。上述多组学证据为影像组学特征提供了生物学注解，被揭示的关键通路也为CIP的靶向治疗提供潜在靶点，推动CIP的精准化治疗；联合多组学构建预测模型，可识别高危患者并实现个体化监测，从而为降低重症CIP发生及疾病死亡率提供证据。

## 4 XAI技术

传统影像组学可解释性强，但性能依赖ROI勾画准确性且特征降维步骤复杂^[44]^。深度学习影像组学采用端到端自动学习特征，性能更优，但“黑箱”性质突出，临床可解释性差^[45]^。XAI技术弥补这一短板：梯度加权类激活映射（gradient-weighted class activation mapping, Grad-CAM）生成热力图，突出显示驱动模型预测的图像区域，局部可解释模型无关解释（local interpretable model-agnostic explanations, LIME）通过局部扰动并训练简单模型为单个预测提供局部解释，而沙普利加性解释（SHapley Additive exPlanations, SHAP）则定量计算特征对预测结果的贡献大小与方向，实现模型全局解释^[46]^。Tan等^[20]^使用类激活映射来可视化深度学习模型的输出，证实两阶段迁移学习将模型的注意力从肺外区域转移到肺内区域，解释了性能的提升。Wang等^[24]^采用Grad-CAM发现胸膜异常也可能提示CIP的发生。与之前采用单一XAI技术的研究不同，Veeramani等^[47]^通过结合改进的U-Net分割方法与LIME和Grad-CAM技术构建了XAI-TRANS模型，可同时呈现对模型决策产生正向或负向影响的可视化特征。XAI技术的核心价值不仅在于“解释”，更在于建立临床医生对人工智能辅助决策系统的信任。当临床工作者能够直观地看到模型“为何”做出某一判断，并确认其判断依据与临床认知一致时，模型才真正具备了融入临床工作流的可能性。因此，未来应进一步扩展XAI技术在疾病影像分析中的应用范围，并开发更稳健、可解释的新技术或联合多种XAI技术提升解释精准度，以支持影像组学模型在临床实践中的应用。表4对3类XAI技术特征进行了比较。

**表4:** XAI技术特征对比

Comparison	LIME	Grad-CAM	SHAP
Category	Model-agnostic	CNN-specific	Model-agnostic
Principle	Instance perturbation	Visual heatmap	Game theory
Output	Local feature contribution	Heatmap	Quantitative contribution values
Stength	High resolution	Intuitive visual heatmap	Global and local interpretation
Limitation	Poor global-interpretation	Limited spatial resolution	High computational cost

XAI: explainable artificial intelligence; LIME: local interpretable model-agnostic explanations; SHAP: SHapley Additive exPlanations.

## 5 研究局限与未来方向

尽管影像组学及其与多组学、XAI技术的融合已在CIP研究中取得显著进展，但该领域仍存在若干共性缺陷，制约了其临床转化进程。现有研究大多为单中心、小样本回顾性设计，不仅影响模型稳定性，还易导致过拟合，使模型泛化能力存疑，凸显出大样本、多中心、前瞻性研究的必要性。此外，当前研究在图像采集、分割、特征提取等方面缺乏标准化方案，影响结果可重复性与多中心验证，亟需建立标准化影像组学特征提取流程，包括统一的扫描参数、明确的ROI勾画区域（全肺、肿瘤病灶、肺实质）以及标准化的特征提取工具。现有模型缺乏与临床信息系统有效对接，且可视化XAI技术的缺失，让临床工作者对模型信任度和采纳意愿受限，未来需开发临床信息-影像-XAI集成智能平台，实现从影像输入、特征提取、风险预测到可视化解释的全流程自动化。CIP是一个动态演变的过程，其影像表现、炎症负荷及临床严重程度均随时间变化。当前研究仅基于基线单时点CT图像构建静态预测模型，未能利用治疗过程中的动态影像资料。已有研究^[43]^初步证实动态影像组学有提高模型鉴别能力和预测效能的趋势，未来研究应进一步挖掘其潜力。

综上所述，影像组学作为一种无创性生物标志物，在CIP的全程综合管理中具有临床转化潜力，但当前研究在标准化流程、外部验证、机制探索及临床转化等方面仍面临重大挑战，仍需我们建立标准化流程、开展前瞻性、多中心验证研究、深化影像组学与多组学数据（病理组学、基因组学、蛋白质组学）的机制研究、发展动态影像组学，并推动影像组学及XAI模型走向临床实践，最终支撑CIP更精准、个体化的诊疗。
